# Correlation between cell-free mRNA expressions and PLGF protein level in severe preeclampsia

**DOI:** 10.1186/s13104-015-1186-9

**Published:** 2015-06-02

**Authors:** Akhmad Yogi Pramatirta, Johannes Mose, Jusuf S Effendi, Sofie Rifayani Krisnadi, Anita Deborah Anwar, Prima Nanda Fauziah, Jeffry Iman Gurnadi, Dwi Davidson Rihibiha

**Affiliations:** Department of Obstetrics and Gynecology, Faculty of Medicine, Padjadjaran University, Bandung, Indonesia; Department of Biology, School of Life Sciences and Technology, Bandung Institute of Technology, Bandung, Indonesia; Department of Biotechnology, School of Life Sciences and Technology, Bandung Institute of Technology, Bandung, Indonesia

**Keywords:** PlGF, Cell-free mRNA PlGF, Preeclampsia

## Abstract

**Background:**

Preeclampsia is a major cause of morbidity and mortality, both maternal and perinatal. The etiology and pathophysiology of preeclampsia remain unknown. Research shows the implantation of the placenta in preeclampsia occurs due to incomplete angiogenic imbalance as one of the preeclampsia pathogenesis. PlGF is angiogenic protein which is synthesized in placenta by mRNA PlGF. When damage occurs, mRNA will be released from cell and form cell-free mRNA. This study aims to analyze the differences between the PlGF mRNA expression in severe preeclampsia and normal pregnancy as well as to measure the relationship between cell-free mRNA and levels of PlGF with the incidence of severe preeclampsia.

**Methods:**

The method used in this study is an observational analytic study with cross-sectional design. Blood samples were obtained from patients with preeclampsia and normal pregnancies as the controlling factors in accordance with inclusion and exclusion criterias. Examination of the PlGF level was measured by ELISA method and mRNA PIGF expression was measured by RT-PCR. Physical and laboratory examinations of patients were recorded and collected as data. Calculations were done by statistical analysis.

**Results:**

Mean of the cell-free mRNA PlGF expression level in severe preeclampsia is 2.2983 ng/mL within the scale of 1.96–2.83 ng/mL and deviation standard of 0.1897. Using Pearson Analysis Test, the result shows that there is a positive correlation between cell-free mRNA expression and PlGF protein level in severe preeclampsia, with r = 0.640 dan p < 0.004.

**Conclusion:**

There is no difference between expression of cell-free mRNA PlGF in severe preeclampsia serum and normal pregnancy. There is a significant correlation between expression of cell-free mRNA and PlGF protein level in severe preeclampsia.

## Background

Hypertension remains a leading cause of maternal mortality and morbidity. Gestational hypertension is defined as hypertension disease which occurs in pregnancy, consisting of: (1) gestational hypertension, (2) preeclampsia and eclampsia syndrome, (3) chronic hypertension which is worsened by preeclampsia, and (4) chronic hypertension. Preeclampsia and eclampsia syndrome are the highest risk among others [1–3]. Preeclampsia and eclampsia syndrome are further called as preeclampsia and eclampsia. Diagnose of preeclampsia is clinically established if there is an increased blood pressure more than 140/90 mmHg and proteinuria is more than 300 mg/24 h (dipstik +1) with or without edema.

Incomplete placental implantation in preeclampsia is caused by angiogenic imbalance as one of pathogenic factors. Angiogenic substance, *placental growth factor* (PlGF) is considered to play its role. PlGF is produced by trophoblast which is responsible in placentation and it is believed to play its role as angiogenic. Studies showed that PlGF has both angiogenic and antiangiogenic properties depending on pathophysiological conditions [4]. The levels of circulatory PlGF increase gradually and peak at mid gestation before declining again in uneventful pregnancies. PlGF concentration profile follows a similar pattern in women who later developed preeclampsia, however with decreased amplitude. Reducing levels of PlGF serum in first trimester causes angiogenesis placenta imbalance, which leads to preeclampsia in second or third trimester [5, 6].

PlGF concentrations are already significantly reduced at the end of the first trimester and remain lower throughout pregnancy. Yet, the difference in circulatory PlGF between normotensive pregnancies and those affected by preeclampsia is the highest within weeks of the onset of the clinical symptoms. As with sFlt-1, the pre-symptomatic levels of circulatory PlGF is thought to associate with the severity or time of onset of preeclampsia [7]. Urinary PlGF is likewise lower in preeclamptic patients before and at the time of symptoms [8, 9].

PlGF protein is produced by ribosome involving DNA to form polypeptide chain via RNA form. It has been reported that there is a circulating fetal mRNA in maternal serum and the role remains unknown. There is one or two such cells per millimeter maternal blood in second trimester pregnancy. They are composed of trophoblast cells, lymphocytes, and nucleated red blood cells (NRBCs). NRBCs originate from fetus carrying fetal cytoplasm and embryonic hemoglobin. However, maternal erythroid precursors containing smaller amounts of these hemoglobins are also present. Paternal DNA sequencing test showed the difference whether blood is derived from fetus or maternal, with 50% specificity in chromosome Y [10].

Circulating fetal cells elevate in Down syndrome and preeclampsia. It has been reported that chromosome-Y sequences could be detected by PCR assay to diagnose cycle-cell anemia and thalassemia B in fetus [11]. Chromosome Y from *cell*-*free fetal* DNA (cff-DNA) was detected by PCR method in serum of pregnant women carrying male fetuses [10, 12]. Source of cff-DNA in maternal circulation remains unclear. It has been hypothesized that cff-DNA originates from fetal cell lysis by the reaction of maternal immune, cell apoptosis during fetus development, trophoblast cell apoptosis and placental aging. Cff-DNA disappears few hours after birth [10].

The value of cff-DNA in maternal plasma as an indicator for preeclampsia has first been reported by Lo et al. in a small scale study in the plasma of 20 preeclamptic women and 20 gestational age matched controls in the third trimester, where cff-DNA was increased approximately fivefold in women with preeclampsia [10]. The same effect in the second trimester was investigated by Zhong et al. in 10 preeclamptic women and 40 controls [13]. The so far biggest study regarding the role of cff-DNA in preeclampsia was conducted by Levine et al. with 120 preeclamptic women and 120 controls: A two to fivefold increase of cff-DNA levels was monitored starting from week 17 until 3 weeks before the onset of preeclampsia [14]. As the amount of fetal DNA is routinely determined by quantifying Y-chromosome specific sequences, e.g. *SRY* (sex determining region Y) and *DYS* [15], alternative approaches have been performed to overcome this limitation: Increased total cff-DNA was observed in women with preeclampsia at term [16–18] and before the onset of preeclampsia [18]. Approaches to analyze cff-DNA independent from fetal sex, using epigenetic differences between maternal and fetal DNA have been developed, e.g. the use of the *maspin* gene, which is hypomethylated in fetal tissue [19] or the hypermethylated fetal promoter sequence of *RASSF1A* [20]. Although these approaches are promising, only one study quantifying cff-DNA with the *RASFF1A* approach in 10 women with preeclampsia and 20 controls has been published [21]. Cff-DNA has shown some predictive value for the prediction of preeclampsia between 20 and 25 weeks of gestation, however, higher sensitivities and specificities can be obtained by combining several markers as has been shown in a nested case–control study for cff-DNA combined with Inhibin A in the second (n = 15 at risk for PE, n = 68 controls) and third trimester (n = 34 preeclampsia, n = 44 controls) [22]. Currently, several multicenter studies are being performed to confirm the predictive value of cff-DNA to predict and monitor preeclampsia in combination with other potential markers, e.g. P-selectin, PAPP-A, PP-13, sFlt-1, sEng, PlGF).

The study of finding the correlation between expression of cff-mRNA and levels of protein in maternal serum is rarely done. The previous studies showed that there is a correlation between cff-mRNA specific gene expression with protein expression in preeclamptic patients, which refers to corticotropin releasing hormone (CRH) gene. This study aims to know the difference between *cell*-*free* mRNA PlGF expression in preeclamptic patients and the correlation with PlGF protein level. It is expected to be one of foundations to understand preeclampsia and fetal-maternal genetic material transfers in placenta.

## Result

Characteristic data, systolic and diastolic blood pressure, measurement of proteinuria, and blood to be measured for PlGF and cff-mRNA PlGF in Prodia laboratory Bandung were collected from both study groups. Characteristics of age, parity, and gestational age were compared for homogeneity.

As shown in Table 1, there is no significant difference (p > 0.05) in age, parity and gestational age between the two groups. Characteristics of subjects are therefore homogenous. Data distribution of PlGF level and cff-mRNA PlGF expression obtained is further examined by Shapiro–Wilk normality test. Results showed that PlGF level and *cell*-*free* mRNA PlGF expression are not normally distributed (p < 0.05). Result of normal distribution obtained was done by conversion analysis to log.

**Table 1:** Characterization of subjects

Characteristic	Group		Total	P value
	Severe preeclampsia (n = 18)	Normal (n = 19)		
1. Age (years old)				
<20	4	1	15	0.303*
21–34	11	15	16	
>35	3	3	6	
x (SD)	27.4 (7.1)	27.0 (5.3)		
Range	17–40	20–36		
2. Parity				
Nulipara	7	6	13	0.195*
Para	11	13	24	
3. Gestational age (week)				
37	8	10	18	0.221**
38	6	2	8	
≥39	4	7	11	
(±SD)	37.1 (2.6)	37.02 (4.3)		
Range	32–41	24–42		

* p value for age and parity was analyzed with Chi Square test.

** p value for gestational age was analyzed with Fisher’s exact test.

Table 2 shows comparison cff-mRNA PlGF expression level between severe preeclampsia and normal pregnancy. There is no change between the two groups (p = 0.744). PlGF protein level in preeclampsia is significantly lower than that in normal pregnancy (p < 0.001).

**Table 2:** Comparison of *cell*-*free* mRNA PlGF expression between PlGF protein level in severe preeclampsia with normal pregnancy

Variable	Group		t test	P value
	PEB (n = 18)	Normal (n = 19)		
Log PlGF				
x (SD)	1.7418 (0.5015)	2.4551 (0.4449)	4.582	<0.001
Range	1–3.13	1.73–3.06		
x p (95/ci)	55.18 (31.07–97.99)	285.17 (174.02–467.20)		
Log mRNA PlGF				
x (SD)	2.2983 (0.1897)	2.325 (0.2906)	0.329	0.744
Range	1.96–2.83	1.96–3.33		
x p (95/ci)	198.75 (159.96–246.94)	211.35 (153.07–291.811)		

**Figure 1:**
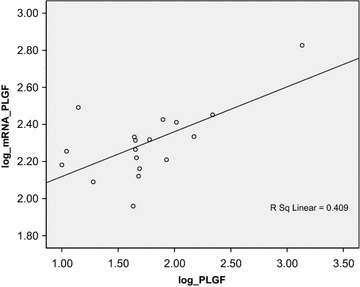
There is correlation between *cell*-*free* mRNA PlGF expression and PlGF protein level in severe preeclampsia. By using log calculation, it showed there is significant correlation between c*ell*-*free* mRNA PlGF expression and PlGF protein level.

There is a correlation between cff-mRNA PlGF expression and PlGF protein level in severe preeclampsia, as shown in Figure 1. Expression of cff-mRNA PlGF is significantly correlated to the levels of PlGF protein which is performed by log calculation. It leads to the theory that mRNA which is located outside cytoplasm is able to be expressed into a protein.

## Discussion

### Characterization of research subjects

This research has 37 subjects involving 18 subjects with severe preeclampsia and 19 subjects with normal pregnancy as controls. Characterization of the subjects in those groups has no significant difference in parity and gestational age.

Group matching was done between severe preeclampsia and controls. Characterization data is homogenous (p > 0.05), as shown in Table 1. There is no significant difference between the two groups which is performed by Chi square test with confidence level of 95%. The characteristics of mothers with p > 0.05 in both groups respectively are; age, p = 0.831; parity, p = 0.195; and gestational age, p = 0.221. It is rare to find such characteristics in cohort studies, especially in finding the predictor of the number of parity and previous obstetric history. Incident of preeclampsia is usually higher in primi or nuli gravida [2, 23]. It is associated to the theory that the first time exposure of villi khorialis is one of the predisposing factors in preeclampsia. Consecutive sampling that may cause differences in parity effect is not significant in this research.

PlGF protein level obtained was analyzed by Shapiro–Wilks test as shown in Table 2. The difference between the results is clearly caused by the small amount of samples.

### Comparison of cell-free mRNA expression in severe preeclampsia and in normal pregnancy

Table 2 shows that there is no difference between cff-mRNA PlGF expression in preeclampsia and normal pregnancy (p < 0.744). However, PlGF protein level is significantly lower in severe preeclampsia than PlGF level in normal pregnancy.

Expression of cff-mRNA PlGF has never been investigated. It is assumed that cff-mRNA PlGF is responsible for PlGF synthesis circulating in maternal circulation. Its roles are similar to other mRNA proteins. Synthesis by mRNA results in certain benefit in angiogenesis in pregnant women. Expression of cff-mRNA PlGF is therefore expected to be comparable with levels of PlGF which is lower in severe preeclampsia than in normal pregnancy. Surprisingly, there is no difference between cff-mRNA PlGF expression in preeclampsia and normal pregnancy as shown in Table 2. Different study designs should be used in checking the validity of the study.

Table 2 shows that there is a significant difference of PlGF level in severe preeclampsia than that of normal pregnancy (p < 0.001). It has been analyzed that PlGF serum level in preeclampsia is lower than in normal pregnancy. This is related to the previous studies of reduced PlGF due to hipoxia placenta in preeclamptic patients [23, 24].

The role of PlGF is not fully understood. It has been reported that PlGF binds with sFlt-1. They play role as angiogenic. However, studies show that PlGF has both angiogenic and antiangiogenic properties depending on pathophysiological conditions. PlGF level elevates in midtrimester in normal pregnancy and it is associated with sFlt-1 level. It is known to be based on the comparison the PlGF level. The role of PlGF level is predominant in the development of the placenta. PlGF is produced in endothelial, trophoblast, monosite and eritroid cells. Hypoxia induces degradation of *Glial Cell Missing* 1 (GCM1), PIGF transcription factor in trophoblast cells, and PlGF production. *Metal*-*responsive transcription factor* (MTF-1) is thought to play role in PlGF synthesis in trophoblast cells. Moreover, MTF-1 reduces when hypoxia is present. However, VEGF and sVEGFR-1 syntheses elevate in hypoxia regulated by hipoxia inducible factor (HIF-1) in placenta. Thus, it leads to overproduction of VEGF and sVEGFR-1 in preeclampsia [25].

PlGF synthesis is possibly generated by VEGF in endothelial cell through mechanism which is associated with protein kinase C and mitogen-activated protein kinase (MEK). PlGF synthesis will reduce due to placental hypoxia and elevated sVEGF-1. Angiogenic levels are extremely low in circulatory which leads to endothelial dysfunction and indicates preeclampsia [25, 26].

### Correlation between cell-free mRNA expression and PlGF Protein level in severe preeclampsia

The cell-free mRNA PlGF expression level is positively correlated with PlGF protein level in PEB group (r = 0.640 dan p < 0.004), as shown in Figure 1. Newly formed mRNA plays role as matrix for protein synthesis (mRNA).

There are only few gene fragments containing genetic code in nucleic cells. Mature RNA is released from nucleus and it binds to ribosome in cytoplasm. RNA is translated in order to match the amino acids sequence (protein) [27, 28]. Placental RNA cells are released from cytoplasm cell. Then, they form a cell-free mRNA in the damage cell such as apoptosis.

Nucleic cells transfer in uteroplacental circulation is reported to undergo in two directions, from mother to fetus back and forth. The transfer causes microchimerism and triggers autoimmune disease in pregnancy. The transfer also occurs in DNA. It has been reported that cff maternal DNA is present in fetus umbilical vein blood, and its number is lower than that in maternal circulation. PIGF protein synthesis in placenta is assumed to be affected by cff-mRNA PlGF in maternal circulation as well.

## Conclusion

There is no difference between expression of *cell*-*free* mRNA PlGF in severe preeclampsia serum and normal pregnancy. However, levels of PlGF serum in severe preeclampsia is significantly lower than in normal pregnancy. There is a significant correlation between expression of *cell*-*free* mRNA and PlGF protein level in severe preeclampsia.

## Methods

This is a cross-sectional analytic observational research. Data are taken once in two groups, both in preeclampsia and in normal pregnancy. Test of PlGF level and *cell*-*free* mRNA PlGF serum expression have been done to those two groups and blood pressure. Edema and proteinuria test have also been done to confirm severe preeclampsia diagnose.

Patients with preeclampsia (18 samples), and normal pregnancies as controls (19 samples) were managed according to the guidelines therapy of Obstetric and Gynecology Faculty of Medicine Padjadjaran University Hasan Sadikin Hospital (FKUP/RSHS), Bandung, through some tests: (1) anamnesis; name, age, address, parity, first day of the last period, gestational age, previous hypertension history, and current pregnancy disease. (2) blood pressure was measured by using sphygmomanometer to the patients who were treated by lying on their left side for 15 min. (3) 6 ml blood sample was taken from peripheral blood before birth, centrifuge at 1,600*g* 10’ 4 °C. Blood sample was withdrawn and then kept in −20 °C temperature. (4) RNA total was extracted from 1.6 mL plasma, homogenized with 2 mL reagent Trizol LS (Invitrogen) and 0.4 chloroform. Suspension was centrifuged at 12,000*g* 15’ 4 °C, aqueous was removed to another tube. Suspension has been added by 1 volume 700 mL/L ethanol, 1 volume aqueous, then added to QIAamp MinElute Virus Column (Qiagen) and processed by manufacture recommend. RNA total was eluted by 20 µL Rnase-free water was directly transcribed. Examination of *cff mRNA* levels was performed by *QIAamp MinElute Virus column* to separate DNA from RNA, TagMan PCR analysis was performed to determine protein. Level of PlGF serum was performed by *high sensitivity indirect sandwich enzyme*-*linked immunosorbent assay* (ELISA).

Data analysis was begun by performing normality test. Categorical data were analyzed with Chi square or Fisher’s exact test when the expected value was less than 5. Normally-distributed data were compared with *t* test, while not-normally-distributed ones were analyzed with Mann–Whitney test. Ratio of proteins was analyzed with Spearman’s rank correlation test. Data analysis was performed with Statistical Package for The Social Sciences software (SPSS) or Windows version 15.0, with 85% confidence interval and p value of <0.05. Written consent were obtained from all participants. The ethical review boards of the Health Research Ethics Committee, the Faculty of Medicine, Padjadjaran University and Dr. Hasan Sadikin Hospital, Indonesia approved this study.
